# Multiple opsins in a reef-building coral, *Acropora millepora*

**DOI:** 10.1038/s41598-023-28476-5

**Published:** 2023-01-29

**Authors:** Benjamin M. Mason, Mitsumasa Koyanagi, Tomohiro Sugihara, Makoto Iwasaki, Vladlen Slepak, David J. Miller, Yusuke Sakai, Akihisa Terakita

**Affiliations:** 1 ARC Centre of Excellence for Coral Reef Studies, James Cook University, Townsville, QLD 4811 Australia; 2 Molecular and Cell Biology, James Cook University, Townsville, QLD 4811 Australia; 3 Department of Biology and Geosciences, Graduate School of Science, Osaka City University, 3-3-138 Sugimoto, Sumiyoshi-Ku, Osaka, 558-8585 Japan; 4 The OCU Advanced Research Institute for Natural Science and Technology, Osaka Metropolitan University, 3-3-138 Sugimoto, Sumiyoshi-Ku, Osaka, 558-8585 Japan; 5 Department of Biology, Graduate School of Science, Osaka Metropolitan University, 3-3-138 Sugimoto, Sumiyoshi-Ku, Osaka, 558-8585 Japan; 6 Department of Molecular and Cellular Pharmacology, University of Miami Miller School of Medicine, Miami, FL USA; 7 Centre for Tropical Bioinformatics and Molecular Biology, James Cook University, Townsville, QLD Australia; 8 Marine Genomics Unit, Okinawa Institute of Science and Technology Graduate University, Onna, Okinawa 904-0495 Japan

**Keywords:** Visual system, Neuroscience, G protein-coupled receptors

## Abstract

Opsins, light-sensitive G protein-coupled receptors, have been identified in corals but their properties are largely unknown. Here, we identified six opsin genes (acropsins 1–6) from a coral species *Acropora millepora*, including three novel opsins (acropsins 4–6), and successfully characterized the properties of four out of the six acropsins. Acropsins 1 and 6 exhibited light-dependent cAMP increases in cultured cells, suggesting that the acropsins could light-dependently activate Gs-type G protein like the box jellyfish opsin from the same opsin group. Spectral sensitivity curves having the maximum sensitivities at ~ 472 nm and ~ 476 nm were estimated for acropsins 1 and 6, respectively, based on the light wavelength-dependent cAMP increases in these opsins-expressing cells (heterologous action spectroscopy). Acropsin 2 belonging to the same group as acropsins 1 and 6 did not induce light-dependent cAMP or Ca^2+^ changes. We then successfully estimated the acropsin 2 spectral sensitivity curve having its maximum value at ~ 471 nm with its chimera mutant which possessed the third cytoplasmic loop of the Gs-coupled jellyfish opsin. Acropsin 4 categorized as another group light-dependently induced intracellular Ca^2+^ increases but not cAMP changes. Our results uncovered that the *Acropora* coral possesses multiple opsins coupling two distinct cascades, cyclic nucleotide and Ca^2+^signaling light-dependently.

## Introduction

Reef-building corals lack eyes and even basic visual structures, yet display notable light sensitivities, including tentacle expansion and retraction^1^ and regulation of reproductive timing^2–4^. Light also influences the behavior of planula larvae which in some species demonstrate phototaxis/photophobic responses^5–7^ and light-dependent selection of settlement substrata^8–10^. The molecular basis of these photoreceptions has been largely unknown.

Thousands of opsins, which are bound to retinal chromophores and form photosensitive pigments, have been identified from various triploblastic animals and they are classified into seven groups^11,12^. Our previous studies have shown that the opsin members belonging to different groups possess different molecular properties, some of which may relate to physiological functions for extraocular photoreception^11,13,14^. In both vertebrates and invertebrates, opsins responsible for extraocular photosensitivity versus ocular photosensitivity are associated with distinct molecular properties and signaling cascades. While coral photosensitivity is inherently non-ocular, multiple opsins with different molecular properties are likely responsible for these various coral photoreceptions.

Opsin genes have been identified in cnidarians including hydrozoans, scyphozoan, cubozoans and anthozoans^15^. Cubozoan opsins have been identified in two box jellyfish species, *Tripedallia cystophora* and *Carybrea rastonii*^16,17^. In corals, three kinds of opsin genes (acropsins 1–3) were identified from cDNA libraries from the species *Acropora palmata*^18^. Together with the genome- and transcriptome-based identification of opsin genes in sea anemones^15,19^, these efforts support the notion that opsins in anthozoans are expressed and have a potential to function.

In the phylogenetic tree of the opsin family, which is composed of several functionally different groups^11^, anthozoan opsins do not form a single cluster, but instead are classified into three distinct ones, suggesting their diversity in molecular properties^19–22^. To date, only box jellyfish opsins have been characterized with respect to spectral characteristics and G protein coupling^17,23^.

Here, we identified six putative opsin genes (acropsins 1–6) in an Indo-Pacific coral, *Acropora millepora*. Phylogenetic analysis indicated that they fall into the three known cnidarian opsin groups, including two anthozoan-specific opsin clades. We heterologously expressed the acropsins in mammalian cells to investigate their molecular properties. Our results demonstrate their sensitivity to blue or UV light and coupling to at least two distinct signaling cascades.

## Results

### Identification of opsins in *Acropora millepora*

**Figure 1:**
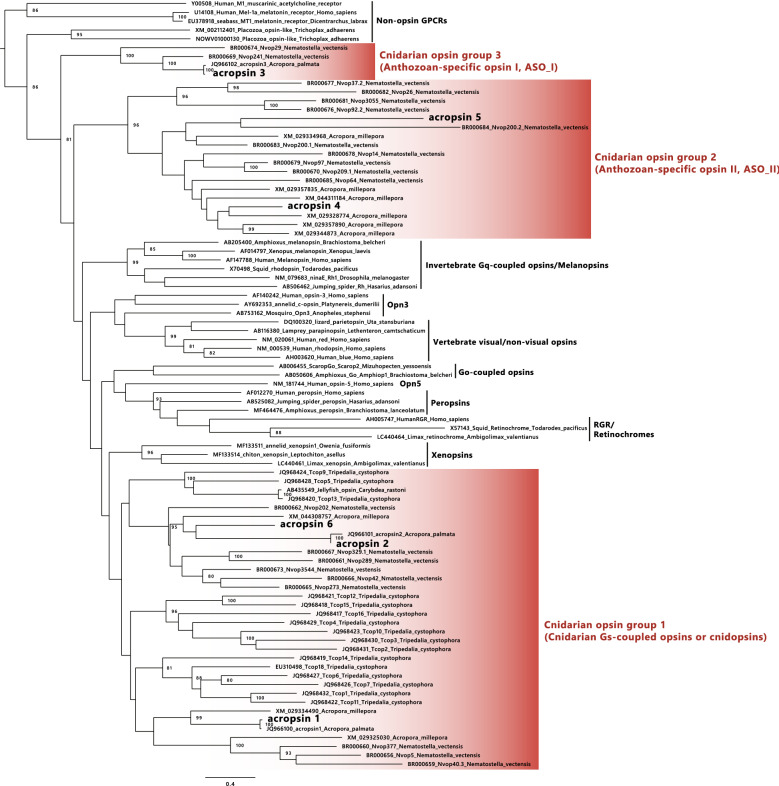
Phylogenetic positions of the six *Acropora millepora* acropsins. The maximum likelihood (ML) tree contains bilaterian, anthozoan and cubozoan visual and non-visual opsin sequences. Numbers at nodes represent support values of ML branch estimated by 1000 bootstrap replicates and the values more than 75% are indicated. Scale bar = 0.4 substitutions per site.

We isolated six cDNAs encoding opsins, acropsins 1–6, from planula larvae of the Indo-Pacific coral, *Acropora millepora*. The deduced amino acid sequences of acropsins had typical features of opsins: seven putative transmembrane domains and the Lys residue for chromophore binding at the seventh transmembrane domain (Lys at position 296, the bovine rhodopsin numbering system). We inferred the phylogenetic tree of the opsin family including acropsins and found that three of the six acropsins in *A. millepora* were orthologs of previously reported *A. palmata* acropsins 1–3^18^, and the other three (acropsins 4–6) were novel coral opsins (Fig. 1). The phylogenetic tree showed that these six genes did not form a single coral opsin clade and fell into three distinct groups corresponding to the previously reported cnidarian opsin clades. Acropsins 1, 2 and 6 fall in the group 1 (cnidarian Gs-coupled opsin group^17^ or “cnidopsin”^24^), acropsins 4 and 5 in the group 2 (“anthozoan-specific opsin II, ASO_II”^15,21^) and acropsin 3 in the group 3 (“anthozoan-specific opsin I, ASO_I”^15,21^).

### Light-induced cAMP increase and spectral sensitivities

**Figure 2:**
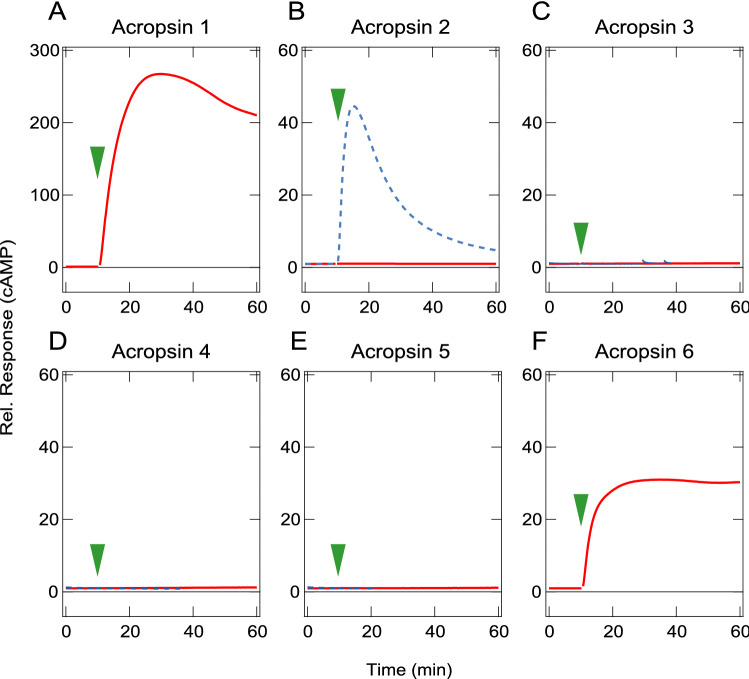
Light-dependent cAMP change in cultured cells expressing acropsins. Intracellular cAMP levels in cultured cells expressing the wild-type (solid red curves) or chimeral mutant (broken blue curves) of acropsins 1–6 (**A**–**F**) were measured as intensity of luminescence signals. Green arrowheads indicate timing of green light (500 nm) irradiations. The luminescence values were normalized to the average baseline during the 60 s immediately preceding irradiation.

**Figure 3:**
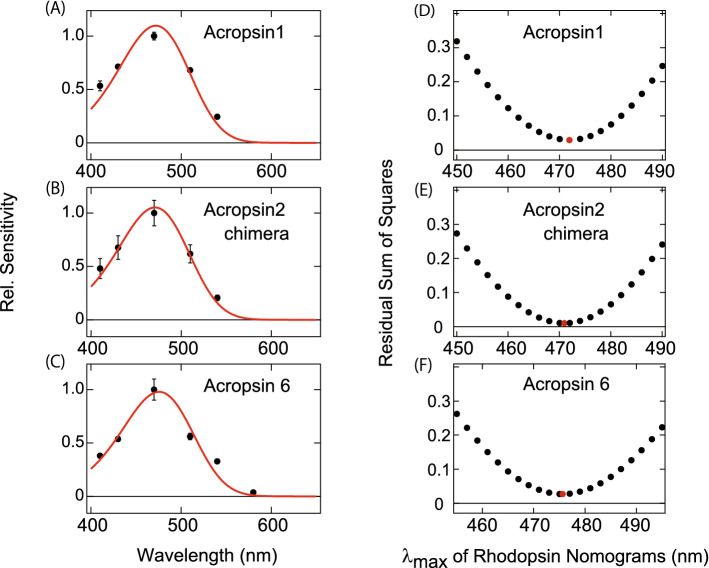
Estimation of the spectral sensitivity curves for acropsins 1, 2 and 6. The spectral sensitivities of (**A**) WT acropsin 1, (**B**) acropsin 2 chimera mutant having the third cytoplasmic loop of the box jellyfish opsin, and (**C**) WT acropsin 6. Black circles represent the mean (n = 3 in **A**–**C**) relative sensitivities (change in luminescence/cAMP) of cultured cells expressing each acropsin at each wavelength of light. Error bars represent standard errors. The spectral sensitivities were fit with the estimated sensitivity curves (Govardovskii templates) with λmax of (**A**) 472 nm, (**B**) 471 nm, and (**C**) 476 nm, respectively (shown as red curves). (**D**–**E**) The values of residual sum of squares (RSS) are shown to assess the goodness of fit between experimentally obtained sensitivities and the sensitivity curves with different λmax values. (**D**) acropsin 1, (**E**) acropsin 2 chimera mutant, and (**F**) acropsin 6. The λmax values of the sensitivity curves for calculating RSS were provided around those of best-fitting curves (shown as red dots) at 2 nm intervals: between 450 and 490 nm for (**D**) and (**E**) or between 455 and 495 nm for (**F**).

Our attempts to obtain absorption spectra of the detergent-solubilized pigments of the recombinant acropsins did not produce interpretable spectra. Therefore, we estimated spectral sensitivities of acropsins by heterologous action spectroscopy (HAS), which is based on light wavelength- and intensity-dependent cAMP increases of opsin-expressing cultured cells^25,26^. We first investigated cAMP change in the cultured cells expressing each of the six acropsins using a cAMP-sensitive luciferase assay. We found a prominent light-induced cAMP increases in the acropsins 1- and 6-expressing cells, suggesting that acropsins 1 and 6 light-dependently activated Gs-type G protein, which is similar to the box jellyfish opsin^17^. In contrast, cells expressing either of the other four opsins did not exhibit any cAMP changes (Fig. 2). We then conducted HAS for acropsins 1 and 6 to estimate their spectral sensitivities (Fig. 3). We calculated relative sensitivities of the acropsin-expressing cells at five wavelengths and fit the relative sensitivity data with the rhodopsin nomogram curve^27^ to estimate a spectral sensitivity curve. The residual sum of squares (RSS) was calculated as a function of λmax of nomogram curves, and the minimum value of residual sum of squares for each acropsin indicates the goodness of fit between the estimated sensitivity curve and sensitivities obtained in the experiments (Fig. 3D,F). The estimated spectral sensitivity curves indicated that acropsins 1 and 6 are blue-sensitive opsins with maximum sensitivities located at approximate 472 nm and 476 nm, respectively (Fig. 3A,C).

We next engineered chimeras for acropsins 2–5 of which wild types exhibited no cAMP changes, with the aim of enabling activation of Gs that leads to large cAMP elevation to estimate spectral sensitivities by HAS. We previously developed a method for investigating the spectral sensitivity of opsins which do not elevate cAMP, that is based on the creation of chimeric proteins with the native third cytoplasmic loop replaced with the third cytoplasmic loop of the Gs-coupled box jellyfish opsin and showed that the replacement of the third cytoplasmic loop did not affect the absorption spectrum of the native opsin^25^. Of the acropsin 2–5 chimeras, light-dependent cAMP increase was only observed for acropsin 2 (Fig. 2B–E). The spectral sensitivity curve of the acropsin 2 chimera obtained by the same methods as the cases of acropsins 1 and 6 exhibited the maximum sensitivity at 471 nm (Fig. 3B,E).

### Light-evoked calcium increase in the acropsin-expressing cells

**Figure 4:**
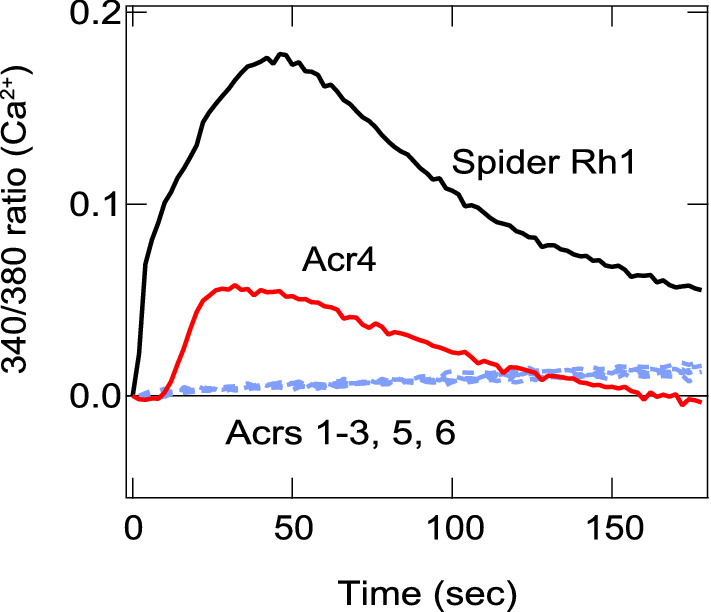
Intracellular calcium increase in the cultured cells expressing acropsins. Light-induced Ca^2+^ increases in the cultured cells expressing each acropsin or jumping spider Rh1 were determined by using the Ca^2+^ fluorescent indicator dye Fura-2. The excitation UV light for Fura-2 was used to stimulate the cells expressing each opsin during the measurement. Note that acropsin 4 (Acr4) elevates Ca^2+^ by a UV light-dependent manner.

Most animal opsins drive cyclic nucleotide- and/or phosphoinositol and free Ca^2+^-mediated phototransduction cascades^17^. Since wild type acropsins 3, 4 and 5 or their chimeras did not exhibit any light-induced cAMP change under the experimental conditions (Fig. 2C–E), we next investigated whether the cultured cells expressing acropsins 3–5 exhibited increases of intracellular free calcium level (Fig. 4). When the cells were exposed to the UV light (340/380 nm) for excitation of Fura-2, we observed a transient increase in Fura-2 fluorescence in the cells expressing acropsin 4 but not in the cells expressing acropsins 3 or 5, although protein expression of acropsins 3 and 5 in cultured cells was observed by immunostaining (Supplementary Fig. S1). Likewise, we did not detect a clear increase of the fluorescence in the cells expressing acropsin 1, 2 or 6. Thus, only acropsin 4 elevated free Ca^2+^ in the cultured cells in a light-dependent manner.

## Discussion

We isolated six opsin genes from the reef-building coral, *Acropora millepora.* The opsins, acropsins 1–6, were phylogenetically divided into 3 groups (Fig. 1). We characterized the some signaling and spectral properties of acropsins 1, 2 and 6 (group 1) and acropsin 4 (group 2). Interestingly, groups 1 and 2 contained the members that drove different signaling cascades leading to increase of cAMP and calcium, respectively (Supplementary Fig. S2). On the other hand, we were unable to observe light-induced cAMP or calcium change for acropsin 3, which belongs to group 3 or acropsin 5 (group 2) although their proteins expression in the cultured cells was detected by immunostaining (Supplementary Fig. S1). The acropsin 3 orthologue in *A. palmata* was shown to light-dependently form a stable product with Gq inducing calcium elevation, by limited trypsinolysis of Gq^18^. This experimental method detecting the distinct biochemical event from the calcium increase and may show different sensitivity to the light-dependent events. Therefore, different methods might have led to the apparently inconsistent results. Further studies for acropsins 3 and 5 are necessary to understand molecular properties of group 2 and 3 opsins.

Acropsins 1, 2 and 6 in the group 1 all had maximum sensitivities in the blue light region. Blue lights are reportedly important for tentacle reaction of adult polyps^1^ and for larval light-responsive behavior^7^ in several scleractinian corals. Furthermore, the nocturnal illumination with blue/green lights has an impact on the spawning timing in *A*. *millepora*^3^. The blue-sensitive acropsins may be responsible for these photosensitivities.

Acropsins 1 and 6 mediated cAMP increase in a light-dependent manner, indicating that they couple to the Gs-type G protein. Together with previous observation that jellyfish opsins elevate cAMP, it is suggested that most opsins in group 1 couple to Gs. However, acropsin 2, which belongs to group 1 did not stimulate cAMP, only its chimeric form did (Fig. 2), suggesting that signaling within this group is not limited to the Gs pathway. Additional experiments will be needed to identify signaling cascades regulated by the members of the cnidarian opsin group 1.

We also showed that acropsin 4 can evoke light-dependent free calcium responses in the cultured cells. To our knowledge, this is the first report to describe signaling of the group 2 opsin. In opsins investigated thus far, protostome visual opsins (Gq-coupled opsins) and deuterostome melanopsin (Opn 4) can elevate calcium level in cultured cells via Gq^28,29^. As acropsin 4 did not show obvious cAMP changes, our findings suggest that it may be the first example of a Gq-coupled opsin outside of the Gq-coupled invertebrate visual opsins and melanopsin group (Fig. 1), although interestingly, it was reported that human Opn5 activate Gq in heterologous cells^30^ whereas mouse and chicken Opn5s activate Gi in the cultured cell and in vitro systems^31,32^. It would also be the first Gq-coupled opsin in a basal metazoan as ancient as the cnidarians and may provide evidence that Gq-coupled opsins evolved in cnidarian and bilaterian lineages independently.

Calcium elevation in the cells expressing acropsin 4 was initiated by UV (340/380 nm) light that is necessary to excite Fura-2. Since UV light is essential for measuring the Ca^2+^read-out, we were unable to investigate the spectral sensitivity of acropsin 4. Interestingly, acropsins 4 and 5 (group 2) have no negatively charged amino acid at the counterion positions, namely sites 94, 113 and 181^33–39^. Because the counterion stabilizes protonation of the retinylidene Schiff base and is essential for the visible light absorption, these characteristics in the amino acid sequences suggest that acropsins 4 and 5 might be UV-sensitive opsins or have the counterion at a position(s) other than the three known positions.

Acropsin 4 belongs to the phylogenetically diverged opsin group from invertebrate Gq-coupled opsin and melanopsin group (Fig. 1) and has a different primary structure from those of the known Gq-coupled opsins. The fact suggests that acropsin 4 might have a unique molecular property as a novel opsin that evokes the intracellular calcium elevation. Optogenetics has made significant contributions to study various biological events involving GPCR^40–42^. Therefore, acropsin 4 might have a potential as a new optogenetic tool controlling free Ca^2+^ signaling, possibly via Gq and an effector enzyme, phospholipase Cβs (PLCβs).

## Materials and methods

### Sample collection

Larval samples of *Acropora millepora* (*A. millepora*) used for RNA isolation were raised in the laboratory from laboratory-crossed gametes. Individual colonies of *A. millepora* were temporarily removed from the inshore reef of Orpheus Island and maintained in flow-through seawater troughs at Orpheus Island Research Station (James Cook University). On the nights of the expected spawning, individual colonies were isolated to keep gametes from individuals separate and equal volumes of the gamete bundles from 5 to 8 colonies were combined to allow out-crossing among individuals. The resulting fertilized embryos were reared in a gentle flow-through seawater system—plankton kriesels (culture tanks with gentle circulation and aeration) and then larvae were collected and frozen for RNA isolation.

### Cloning of opsins

Using *A. palmata* opsin sequences reported previously^18^ as queries, we searched for opsin sequences in in-house transcriptome data for *A. millepora* and found six opsin sequences. Based on the sequences, we obtained the full-length cDNAs of five opsins (acropsins 1, 2, 4–6) and the partial cDNA of acropsin 3 (which has the shortened C terminal tail relative to *A. palmata* acropsin 3^18^) by RT-PCR. Briefly, total RNA was isolated, using a TRIzol (Invitrogen), from *A. millepora* planulae (~ 100 larvae). The cDNAs were synthesized from the total RNA by reverse transcription using Superscript II Reverse Transcriptase (Life Technologies) and were applied to PCR reaction as a template.

### Phylogenetic tree inference

We first selected representative opsin sequences belonging to eight functional groups (i.e., vertebrate visual/non-visual opsin, Opn3, Go-coupled opsin, cnidarian Gs-coupled opsin (cnidopsin), Opn5, invertebrate Gq-coupled opsin/melanopsin, peropsin and RGR/retinochrome^11^) and xenopsin, which is a recently characterized subgroup of opsin^22^. To identify candidate opsin sequences of two anthozoan species (*Nematostella vectensis* and *Acropora millepora*), we carried out BLASTP search against the available genome database (NCBI RefSeq, accession numbers: GCA_000209225 for *N. vectensis*; GCA_013753865.1 for *A. millepora*) with an e-value cutoff of 1e-10 using the amino acid sequence of bovine rhodopsin (accession number: AH001149) as a query. We aligned the candidate sequences and removed sequences that lacked the conserved lysine residue homologous to Lys-296 of bovine rhodopsin. Candidate opsin sequences of *Nematostella vectensis* were compared with the previously published opsin data set^19,20^, and redundant duplicates and fragmented sequences were also removed. We added acropsin sequences (acropsin 1–6) cloned in this study, the putative opsin sequences of *Nematostella vectensis* and *Acropora millepora* and sequences of melatonin receptors, a muscarinic acetylcholine receptor and Placopsins to the representative opsin dataset. The final set of sequences was aligned using MAFFT version 7.2^43^ and gaps in the aligned sequences were removed using trimAl version 1.2^44^ with the ‘gappyout’ option. The maximum likelihood (ML) tree was reconstructed using RAxML-NG version 1.1^45^ assuming the LG + G4 + FC model, which was selected based on AICc using ModelTest-NG version 0.2.0^46^. ML branch supports were estimated with 1000 bootstrap replicates.

### Spectral sensitivity based on GloSensor assay

Heterologous action spectroscopy based on changes in the intracellular cAMP level of opsin-expressing HEK293S cells was performed using the GloSensor cAMP assay (Promega), as described previously^25,26^. Briefly, the opsin expression vectors were transfected into HEK293S with the pGloSensor-22F cAMP plasmid (Promega) using the polyethyleneimine (PEI) transfection method. The transfected cells were incubated overnight at 37°C and after supplementation of 11-*cis* retinal, cells were further incubated overnight at 37°C. Before measurements, the culture medium was replaced with a CO_2_-independent medium containing 10% FBS and GloSensor cAMP Reagent stock solution (Promega). Luminescence derived from GloSensor, an indicator of intracellular cAMP, was measured at 25°C using a GloMax 20/20n Luminometer (Promega). The cells were illuminated with light-emitting diode (LED) light for 5 s and the light-induced changes in luminescence were measured. The measured luminescence values were normalized to those just before the irradiations. LEDs with spectral emission peaks of 410, 430, 470, 510, and 540 nm arrayed on a board (SPL-25-CC; REVOX Inc., Kanagawa, Japan) were used as light sources for measurements of wavelength-dependent responses of opsin-expressing cultured cells. The quantum flux of each LED light was adjusted to 6.2 * 10^14^ or 2.2 * 10^14^ photons/cm^2^/s using interference filters (MZ0410, MZ0430, MZ0470, MZ0510 and MZ0540; Asahi Spectra Co., Ltd.), neutral-density (ND) filters (SIGMAKOKI Co., Ltd., Saitama, Japan and Shibuya Optical Co., Ltd., Saitama, Japan) and ground-glass (Shibuya Optical Co., Ltd.).

Dose (intensity)-response curves were generated for cultured cells expressing each of the opsins by irradiating cells with blue (470 nm) LED light (Ex-DHC; Bio Tools Inc. Gunma, Japan) at 5 different intensities, established using a series of neutral-density (ND) filters. It should be noted that individual dishes of cells were irradiated only once during the measurements, and at least three independent measurements were made at each wavelength or intensity. The intensity–response curve was obtained by fitting a sigmoid function (V = Vmax * In/(In + Kn), where V is the response amplitude, Vmax is maximum response amplitude, I is the stimulus light intensity, K is stimulus intensity eliciting 50% Vmax, and n is the exponent) to the mean responses at each intensity of light irradiation.

The amplitude of the wavelength-dependent responses was extrapolated to the intensity-response curve to transform the amplitude into photon numbers required for the responses. Absorption spectra were estimated by fitting a rhodopsin template^27^ to the relative sensitivities according to the least squares method with the aid of IGOR Pro software (WaveMetrics).

### Calcium imaging assay

Calcium imaging assay was performed as described elsewhere^29^. Briefly, HEK293S cells were transfected with a plasmid DNA by PEI transfection method. Five hours after the transfection, retinal was added to the transfected cells, which were then incubated in Krebs–Ringer HEPES buffer containing 5 μM Fura 2-AM (Dojindo, Japan) next day of the transfection. Fura-2 fluorescence was measured using a fluorescence microscope (BX-51, Olympus) with a CMOS camera (ORCA-Flash4.0, HAMAMATSU) and MetaMorph software (Molecular Devices).

### Accession numbers

Acropora millepora Acropsin 1, MK829324; Acropsin 2, MK829325; Acropsin 3, MK829326; Acropsin 4, MK829327; Acropsin 5, MK829328; Acropsin 6, MK829329.

## Supplementary Information

Supplementary Information.

## Data Availability

The datasets used and/or analysed during the current study available from the corresponding author on reasonable request.
