# Supplementary material for: Multiple opsins in a reef-building coral, Acropora millepora
Source: Sci Rep. 2023 Jan 29;13:1628. doi: 10.1038/s41598-023-28476-5 (PMC9884665; doi:10.1038/s41598-023-28476-5)
Supplement: Supplementary file 1 — Supplementary Information. [file 41598_2023_28476_MOESM1_ESM.docx]

Supplementary Information for

**Multiple opsins in a reef-building coral, *Acropora millepora*.**

Authors: Benjamin M. Mason^1, 2^ *, Mitsumasa Koyanagi^3, 4, 5^*, Tomohiro Sugihara^3, 5^, Makoto Iwasaki^5^, Vladlen Slepak^6^, David J. Miller^7, 8^, Yusuke Sakai^5^,

Akihisa Terakita^3, 4, 5^

*equally contributed

^1^ ARC Centre of Excellence for Coral Reef Studies, James Cook University, Townsville, 4811, Queensland, Australia

^2^ Molecular and Cell Biology, James Cook University, Townsville, 4811, Queensland, Australia

^3^Department of Biology and Geosciences, Graduate School of Science, Osaka City University, 3-3-138 Sugimoto, Sumiyoshi-ku, Osaka 558-8585, Japan.

^4^The OCU Advanced Research Institute for Natural Science and Technology, Osaka City University, 3-3-138 Sugimoto, Sumiyoshi-ku, Osaka 558-8585, Japan.

^5^ Department of Biology, Graduate School of Science, Osaka Metropolitan University, 3-3-138 Sugimoto, Sumiyoshi-ku, Osaka 558-8585, Japan.

^6^ Department of Molecular and Cellular Pharmacology, University of Miami Miller School of Medicine, Miami FL, USA

^7^ Centre for Tropical Bioinformatics and Molecular Biology, James Cook University, Townsville, Queensland, Australia

^8^ Marine Genomics Unit, Okinawa Institute of Science and Technology Graduate University, 904-0495, Onna, Okinawa, Japan

**Supplementary Text**

**Materials and methods**

Checking acropsin expression in the cultured cells by immunostaining.

Protein expression of acropsin 1, 3 and 5 were detected by immunostaining using the rho 1D4 antibody [1] that recognize the epitope sequence (ETSQVAPA) tagged to the C-terminus of acropsins according to the previous report [2]. The HEK 293 Cells expressing acropsin 1, 3 or 5 were fixed in 100% methanol for 5 min, treated with phosphate buffered saline containing 2% bovine serum albumin and 0.1% Tween-20 for 30 min at room temperature and incubated with rho1D4 (hybridoma culture fluid) overnight at 4°C. Subsequently, cells were incubated with Alexa Fluor 488-conjugated anti-rabbit IgG (diluted 1:500; Invitrogen).

References

1. MacKenzie, Arendt, Hargrave, McDowell, Molday: **Localization of binding sites for carboxyl terminal specific anti-rhodopsin monoclonal antibodies using synthetic peptides**. *Biochemistry*, 1984, 23, 6544-6549.
2. Kawano-Yamashita, Koyanagi1, Wada, Saito, Sugihara, Tamotsu, Terakita: **The non-visual opsins expressed in deep brain neurons projecting to the retina in lampreys.** *Sci Rep,* 2020, **10**, 9669.


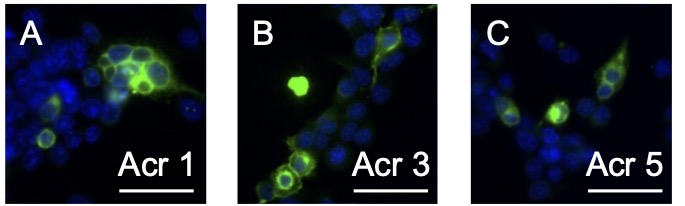


C

A

B

**Supplementary Figure S1 Immunostaining of the cultured cells transfected with acropsin plasmids.**

Protein expression of acropsin 1 (A), 3 (B) and 5 (C) (green) were detected by immunostaining using the rho 1D4 antibody that recognize the epitope sequence (ETSQVAPA) tagged to the C-terminus of acropsins. The signals were visualized by immunofluorescent detection using Alexa Fluor 488 anti-mouse IgG (Thermo Fisher Scientific). The signals of cell nuclei (blue) were visualized by the Hoechst staining (Hoechst 33258). The scale bars represent 50 μm.


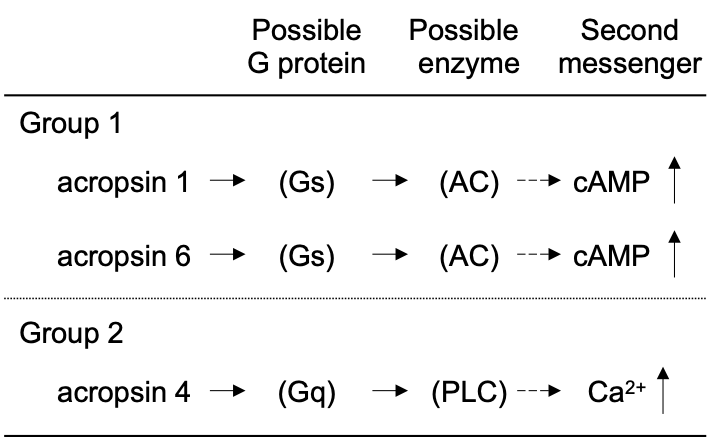


**Supplementary Figure S2** **Diagrams of coral opsin-mediated downstream pathways.**

Possible signaling cascades driven by light-activation of acropsin 1, 6 and 4. AC, adenylyl cyclase, PLC, phospholipase C.
